# Supplementary material for: Predicting visual function by interpreting a neuronal wiring diagram
Source: Nature. 2024 Oct 2;634(8032):113–23. doi: 10.1038/s41586-024-07953-5 (PMC11446822; doi:10.1038/s41586-024-07953-5)
Supplement: Supplementary file 6 — CRF and ERF predictions for individual TmY4 and TmY9 cells. Analogous to Supplementary Data 3, but for TmY target types. Shown are the top four monosynaptic pathways, the strongest pathway passing through each of the top ten intermediary types (ranking from Extended Data Fig. 7), and the trisynaptic pathway Tm1–TmY–Dm3–TmY (see the section entitled Prediction of spatial normalization). [file 41586_2024_7953_MOESM6_ESM.zip › DataS4/TmY9qΓèÑ/720575940625836692.pdf]

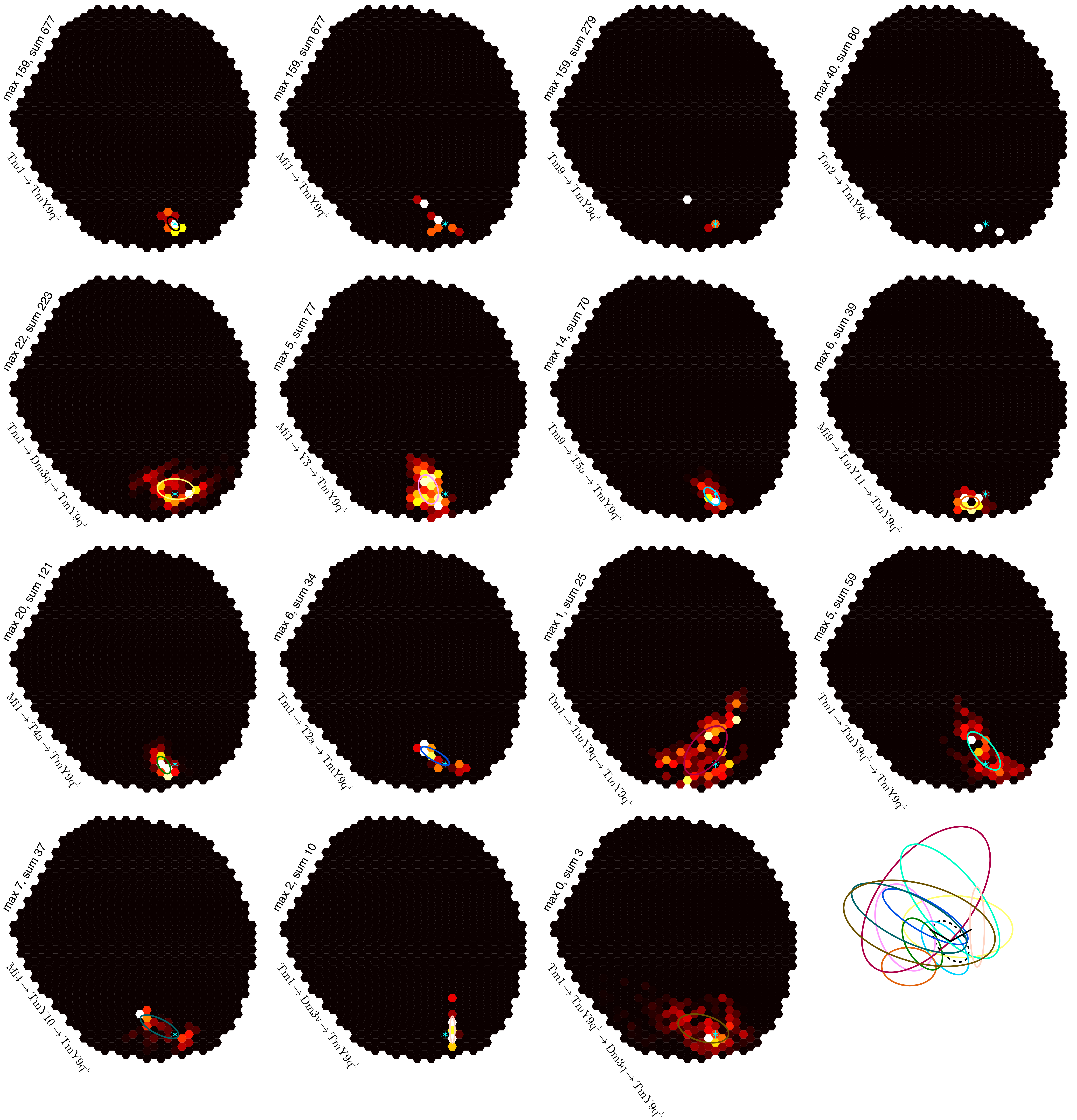

max 159, sum 677
Tm1 → TmY9q⊥
max 159, sum 677
Mi1 → TmY9q⊥
max 159, sum 279
Tm9 → TmY9q⊥
max 40, sum 80
Tm2 → TmY9q⊥
max 22, sum 223
Tm1 → Dm3q → TmY9q⊥
max 5, sum 77
Mi1 → Y3 → TmY9q⊥
max 14, sum 70
Tm9 → T5a → TmY9q⊥
max 6, sum 39
Mi9 → TmY11 → TmY9q⊥
max 20, sum 121
Mi1 → T4a → TmY9q⊥
max 6, sum 34
Tm1 → T2a → TmY9q⊥
max 1, sum 25
Tm1 → TmY9q → TmY9q⊥
max 5, sum 59
Tm1 → TmY9q⊥ → TmY9q⊥
max 7, sum 37
Mi4 → TmY10 → TmY9q⊥
max 2, sum 10
Tm1 → Dm3v → TmY9q⊥
max 0, sum 3
Tm1 → TmY9q⊥ → Dm3q → TmY9q⊥
